# Discrete Affective States, Cortisol, and Self-Rated Health in Old Age

**DOI:** 10.1093/geroni/igab046.2201

**Published:** 2021-12-17

**Authors:** Theresa Pauly, Oliver Schilling, Ute Kunzmann, Hans-Werner Wahl, Nilam Ram, Denis Gerstorf, Christiane Hoppmann

**Affiliations:** 1 University of Zurich, University of Zurich, Zurich, Switzerland; 2 University of Heidelberg, Heidelberg, Baden-Wurttemberg, Germany; 3 Leipzig University, Leipzig, Sachsen, Germany; 4 Heidelberg University, Heidelberg, Baden-Wurttemberg, Germany; 5 Stanford University, Stanford, California, United States; 6 Humboldt University of Berlin, Humboldt University of Berlin, Brandenburg, Germany; 7 University of British Columbia, University of British Columbia, British Columbia, Canada

## Abstract

Old age is a developmental phase in which physical vulnerability increases and discrete affective states are uniquely important. The current project combines data from four studies (total N = 476 participants) to investigate within-person fluctuations in salivary cortisol (a marker of physiological arousal), seven discrete affective states, and the moderating role of self-rated health. Each participant provided affect reports and collected salivary cortisol 5-7 times a day for a 7-day period, and rated their health status. Multi-level models showed that cortisol levels were decreased in moments when participants felt happier, more relaxed, and more interested than usual and increased in moments when participants felt angrier, more nervous, more overwhelmed, and sadder than usual. Associations of happy, nervous, overwhelmed, and sad with cortisol were more pronounced in participants of better as compared to those of worse self-rated health. Findings suggest that higher HPA reactivity may indicate preserved health in older adults.

